# P81 - Clinical analysis of health education and self management combined with drug therapy in school-age children with asthma

**DOI:** 10.1186/2045-7022-4-S1-P136

**Published:** 2014-02-28

**Authors:** Yuanqing Chen

**Affiliations:** 1 Child Health Care, Women and Children Health Institute, Futian, Shenzhen, China

## Objective

To evaluate the effects of health education and self management intervention combined with drug therapy in school-age children with asthma.

## Methods

320 cases of school-age children with asthma were divided into management group and control group, two groups of children were receiving standardized drug inhale treatment, management of children receive regular health education and self management behavior intervention.

## Results

Times of asthma attacks, emergency and missing school days decreased significantly compared with the control group. Pulmonary function improved.

## Conclusion

The health education and self management behavior intervention combined with drug therapy can improve the level of understanding of disease in children with asthma, medication compliance, reduce symptoms,improve lung function and improve the quality of life.

